# Metastatic Breast Cancer to the Gastrointestinal Tract: Report of Five Cases and Review of the Literature

**DOI:** 10.1155/2012/439023

**Published:** 2012-10-04

**Authors:** Massimo Ambroggi, Elisa Maria Stroppa, Patrizia Mordenti, Claudia Biasini, Adriano Zangrandi, Emanuele Michieletti, Elena Belloni, Luigi Cavanna

**Affiliations:** ^1^Department of Oncology and Hematology, Oncology Unit, Azienda Ospedaliera “Guglielmo da Saliceto”, Via Taverna 49, CAP 29121 Piacenza, Italy; ^2^Department of Pathology, Azienda Ospedaliera “Guglielmo da Saliceto”, Via Taverna 49, CAP 29121 Piacenza, Italy; ^3^Department of Radiology, Azienda Ospedaliera “Guglielmo da Saliceto”, Via Taverna 49, CAP 29121 Piacenza, Italy

## Abstract

Luminal gastrointestinal (GI) metastases from breast cancer are rare, reports are fragmentary and poor. The purposes of this study are to assess the gastrointestinal involvement from breast cancer in a retrospective study at a single institution and reviewing the related literature. Between January 2007 and December 2011 a total of 980 patients with breast cancer were treated at our institution, patients' records and report database were analysed. Institutional Review Board approval was obtained for this study. A search of the literature using PubMed, CancerLit, Embase, was performed. Selected for the present review were papers published in English before June 2012. Five of 980 patients (0.5%) showed gastrointestinal metastases from breast cancer, 3 patients had gastric involvement, 1 jejunum, and 1 rectum. Reviewing the literature, 206 patients affected by gastrointestinal metastasis from breast cancer were identified: the most frequent site of metastasis was the stomach (60%). The majority of the patients underwent chemotherapy and endocrine therapy, someone surgery and radiotherapy. GI metastases from breast cancer are rare, but possible, and a very late recurrence can also occur. Cyto-histological diagnosis is mandatory, to differentiate GI metastases from breast cancer to other diseases and to allow an adequate treatment.

## 1. Introduction 

Breast cancer is the most common tumor in women; one in eight women will be affected in their lifetime [1]. Common sites of breast cancer metastasis include lungs, liver, bones, soft tissue, brain, and adrenal glands.

Gastrointestinal (GI) tract metastases from breast origin are considered rare in clinical practice [2]; however, the occurrence in autopsy series varied from 8% to 35% [3, 4]. Most series report a greater propensity for lobular carcinoma to metastasize to the GI tract [5]; reports on this subject in the literature are poor and mostly limited to case reports [6].

Since metastases to GI from breast cancer are uncommon and peculiar, the main problem is to recognize them in patients affected by breast cancer and with GI symptoms, like nausea and vomiting, diarrhoea, and abdominal pain. These symptoms may be considered treatment related or secondary to other GI diseases, or to peritoneal carcinomatosis, and this can delay the definite diagnosis and treatment of GI involvement by breast cancer. 

We report here a series of 5 patients affected by GI metastases from breast cancer, and we reviewed the related literature.

## 2. Patients and Methods

A retrospective analysis was conducted on the records of the patients with breast cancer admitted and treated at the Oncology-Hematology Department, Hospital of Piacenza, Italy, from January 2007 to December 2011; five patients with gastrointestinal metastases from breast cancer were identified, and they form the basis of this report. The medical records were reviewed for presenting symptoms, concurrent metastatic sites, treatment, and followup. Pathology of the GI involvement as well as the primary tumor was reviewed by an expert pathologist in breast cancer. Histologic grading, estrogen (ER) and progesterone (PR) receptor expression, epidermal growth factor receptor-2 (HER2) status, and proliferation rate (ki-67) were done in the sample of primary carcinoma and in the GI metastasis. Original breast cancer presentations were staged according to the 2006 American Joint Committee on Cancer Staging system. 

## 3. Review of the Literature

A computerized literature search through Medline, Cancerlit, and Embase was performed applying the words: breast cancer, gastrointestinal metastases, gastrointestinal tract, and palliative surgery. Articles and abstracts were also identified by backreferencing from original and relevant papers. Papers published in English before June 2012 were Selected for the present review.

## 4. Case Reports and Results from the Literature

During the 5-year study period, 980 patients were diagnosed with breast cancer at our institution. Five of these 980 patients (0.5%) had metastatic disease to the GI tract. Patients' characteristics are described below and summarized in Table 1; the results of literature review are reported in Table 2.

### 4.1. Case  1

A 47-year-old female underwent at another institution left breast mastectomy in March 1985 for a ductal infiltrating carcinoma (DIC) pT2a N0/12. A complete staging was performed, with abdominal ultrasound, chest radiography, and bone scan; no metastases were found. No adjuvant therapies were done.

In February 2006 she showed relapse with left infraclavicular mass and left arm lymphedema.

The histological biopsy of the mass and histological revision of the previous mastectomy showed ER >95% PR 0% Ki-67 10%. She underwent local radiotherapy, with complete response, and endocrine therapy with anastrozole 1 mg/day.

In September 2007 the patient was admitted to our department, since she developed fatigue and anemia: laboratory tests showed anemia, Haemoglobin 7 g/dL, and the fecal occult blood test was positive; an esophagogastroduodenoscopy (EGDS) and a colonescopy were performed: the first one was unremarkable, and the second one discovered the presence of a 4 mm tubule-villous adenoma with mild dysplasia of the right colon, that was removed. She was transfused with 4 units of red blood cells.

In November 2007 the symptoms and anemia recurred (Haemoglobin 6.5 g/dL). The patient was transfused with 3 units of red blood cells, an enteroscopy with videocapsule was performed; an ulcerated and stenosing neoplasm, localized between the duodenum and the jejunum, was diagnosed. Subsequently, a chest-abdominal computed tomography (CT) scan was performed, which revealed absence of lung and liver metastases. 

The patient underwent surgery, with jejunum resection, and the histological examination showed jejunum metastasis from breast cancer, ER 70% PR 0% HER2 negative, like the primary tumor.

Anastrozole was stopped and a treatment with chemotherapy: 5-fluorouracil, epirubicin, and cyclophosphamide (FEC regimen), was started, and continued for 6 cycles. Reevaluation CT scan performed in July 2008 resulted negative. From September 2009 endocrine therapy with Exemestane was started.

The patient is alive and is treated with weekly paclitaxel, since she showed subcutaneous thoracic relapse in July 2011.

### 4.2. Case  2

A 40-year-old woman in May 2008 developed left breast cancer, ductal infiltrating carcinoma with signet ring cells G2 ER 90% PR 35% Ki-67 15% HER2 negative, diagnosed with Tru-cut biopsy; in addition, ultrasound showed 2 pathologic lymph nodes in the left axilla; at this time a colonscopy, performed for rectal bleeding, showed a substenosis 7 cm from the anal margin, biopsies diagnosed metastasis from breast cancer, moderately differentiated (G2) carcinoma, with partial aspects of signet ring cells, ER 85% PR 30% HER2 negative. Chest X-ray, abdominal ultrasound, and bone scan showed absence of metastases.

She was treated with epirubicin (75 mg/m²) and docetaxel (75 mg/m²) days 1–21, 6 cycles, with stable disease.

In November 2008 endocrine therapy with tamoxifen 20 mg/day and LH-RH analogue 11.25 mg every 3 months was started, and symptomatic radiotherapy on the rectum was planned.

Ten months later, patient showed progression in the rectum and liver and was treated with liposomal doxorubicin (50 mg day 1–21) and carboplatin (AUC 5 = 300 mg day 1–21). After 3 cycles she showed disease progression, and chemotherapy with oxaliplatin, folinic acid and 5-fluorouracil was initiated. 

After 12 cycles of chemotherapy she showed gradual improvement with partial response. Patient is now well, with stable disease, and is treated with endocrine therapy, tamoxifene 20 mg/day.

### 4.3. Case  3

A 53-year-old woman in 1987 underwent left mastectomy for breast cancer at another institute, and then no other therapy was performed. 

In October 2007, patient was admitted to our department, since she had pain in the right chest, a bone scan was performed, and multiple bone metastases were detected. The reviewed histological specimen of the previous mastectomy showed DIC G2 ER >80% PR >80% Ki-67 10% HER2 negative. A CT scan of chest, abdomen, and pelvis confirmed multiple bone lesions without other metastases.

The patient was treated with bisphosphonates and letrozole 2.5 mg/day.

Since the patient had dyspepsia, in March 2008, EGDS with biopsies was performed revealing the presence, in the fundus, of carcinoma with solid-chord structure, whom morphology and immunophenotype (G2, ER 70% PR 60% HER2 negative) were coherent with origin from the breast cancer.

In April 2008, weekly chemotherapy with epirubicin and cyclophosphamide was started. After 2 months of treatment, she showed disease progression and underwent a chemotherapy with capecitabine and vinorelbine, but the patient died in August 2008 with disease progression.

### 4.4. Case  4

A 57-year-old women underwent, in January 2007, a quadrantectomy of the right breast and axillary dissection for lobular infiltrating carcinoma (LIC) pT2 N3a (17/20) ER 80% PR 85% Ki-67 10% HER2 negative. Staging detected bone metastases.

Epirubicin (75 mg/m²) and docetaxel (75 mg/m²), days 1–21, chemotherapy was performed for 6 cycles, with partial response. Endocrine therapy with letrozole 1 cp/die was then initiated. After 1 year she had disease progression in the bone, so a new chemotherapy regimen with liposomal doxorubicin 50 mg/m² days 1–21 was initiated.

After 4 cycles, since tumour markers CEA and CA 15-3 were increasing, liposomal doxorubicin was stopped after four cycles and endocrine therapy with fulvestrant 250 mg 1 fl i.m. per month was initiated.

In November 2008, she showed persistent inappetence, nausea, and vomiting, an EGDS with biopsies revealed the presence in the cardia of metastasis from breast cancer (ER 70%, PR 65%, Ki-67 15%, HER2 negative).

In January 2009 capecitabine 1200 mg/m² bid days 1-14q21 was initiated, but after 2 cycles the treatment, was stopped for haematological, gastrointestinal toxicity and disease progression.

The patient died in May 2009.

### 4.5. Case  5

A 63-year-old women underwent in 1998 a radical mastectomy of the right breast and axillary dissection for lobular infiltrating carcinoma (LIC) pT2 N2 ER 50% PR 0% Ki-67 10% HER2 negative. A complete staging was performed, with abdominal ultrasound, chest radiography, and bone scan; no metastases were found. Epirubicine (90 mg/m²) and cyclophosphamide (900 mg/m²), days 1–21, chemotherapy was performed for 6 cycles as adjuvant therapy followed by endocrine therapy with tamoxifen 20 mg/die for five years.

Twelve years later, in October 2010, patient was hospitalised for severe fatigue, inappetence, nausea, anorexia, and bone pain. A total body CT scan and magnetic resonance imaging (MRI) of the abdomen showed liver metastases and omogeneous thickening of the gastric wall. EGDS with biopsies was performed revealing the presence of carcinoma with solid-chord structure, whom morphology and immunophenotype were coherent with origin from the breast (ER 40% PR negative and HER2 negative). Bone scan showed multiple bone metastases. Patient is in treatment with bisphosphonates and letrozole 2.5 mg/day. She also underwent symptomatic radiotherapy on the pelvic bone. 

She is alive, showing gradual improvement of general conditions.

### 4.6. Results from the Literature Review

After reviewing the literature, we found 206 patients reported with GI tract involvement from breast cancer, from 1943 to June 2012. Each part of the GI tract has been reported involved, from the tongue to the anum [1–86].

Site of metastases, presentation's symptoms (a patient could have more symptoms or more sites of metastasis), and other clinical data are reported in Table 2.

- 125 patients had metastases to the stomach (60%), 57 presenting with linitis plastica, 27 with obstruction/stenosis, 12 with ulcer erosion/bleeding, 11 with dyspepsia, 10 with pain as the main symptom, 2 with polypoid lesions, 2 with perforation, and in 4 cases presentations' symptoms were not reported [1, 2, 27–48].
- 24 patients had metastases to the esophagus (12%), 20 presenting with stricture, 3 with achalasia, and 1 with nonspecific dysmotility [2, 8–26].
- 23 patients had metastases to the colon (11%), 16 presenting with obstruction/stenosis, 2 with multiple strictures/diffuse infiltration, 2 with polypoid lesions, 1 with linitis plastica, 1 with asymptomatic abdominal mass, and in 1 case presentations' symptoms were not reported [1, 2, 47, 53, 54, 61–72].
- 16 patients had metastases to the small intestine (8%), 12 presenting with obstruction/stenosis, 1 with multiple strictures/diffuse infiltration, 1 with peritonitis, 1 with bleeding, and in 1 case presentations' symptoms were not reported [2, 49–60].
- 14 patients had metastases to the rectum (7%), 11 presenting with obstruction/stenosis, 1 with linitis plastica, 1 with abdominal pain, 1 with rectal bleeding [33, 39, 46, 56, 61, 73–77].
- 2 patients had metastases to the oropharynx (1%), 1 presenting with oropharyngeal dysphagia, and 1 with a tongue lesion [2, 7].
- 2 patients had metastases to the anum (1%), 1 presenting with polypoid lesion, and in 1 case presentations' symptoms were not reported [78, 79].

In many reports data on treatment are lacking. The majority of the patients underwent chemotherapy and endocrine therapy, someone surgery, and few patients radiotherapy. In one case with esophageal involvement, esophageal dilatation was performed for stricture [2]. In one case, only palliative care was done [2].

## 5. Discussion

Breast cancer is the most common cancer in females and metastasizes frequently to lung, pleura, skin, bone, soft tissues, liver, surrenal gland and brain. The GI tract is an uncommonly reported site of metastatic disease [27]. After reviewing the literature, except a series of 51 cases concerning a 20-year period [27], reports on this subject are poor and often limited to single case reports.

Spread to GI tract seems to be more frequent in lobular histology; the reason is unknown, but some authors think that it could be related to a particular tropism of lobular cells [2, 80]. Pectasides et al. [28] reported, from 1995 to 2008, 8 cases of gastric metastases from breast cancer; the majority of the tumors had lobular histology. It must be emphasized that this localization can simulate a primary gastrointestinal cancer, and any region of GI tract can be involved, from the tongue [29] to the anum [78]. 

The most common type of gastric involvement is as “linitis plastica” appearance due to diffuse intramural infiltration of the stomach by the tumor; this is characterized by a narrowing of the stomach lumen, rigidity, and diminished peristalsis, as reported in our case 5. Other appearance of gastric metastases includes nodular or polypoid lesions and ulcerated lesions [30] CT scan can be useful for noninvasive assessment for estimation of the degree of tumor penetration through the gastrointestinal wall as demonstrated in our patient no. 5. With modern multiphase, multidetector spinal CT imaging, there is increased accuracy in the assessment of mural penetration [87, 88]. 

Metastases to GI tract can be the first manifestation of breast cancer metastases, as well as it can represent relapse even after many years from the diagnosis of the primary tumor, as in our case  3. Surgery is often needed for diagnosis and treatment of complications, like intestinal obstruction or bleeding. 

The most common site of GI involvement from breast cancer observed in our series, like in the literature, was the stomach (3 cases), while in 1 case the disease was localized to the jejunum, and in 1 to the rectum. Colon involvement is quite common, while small intestine involvement is very rare and diagnosed very late, frequently at autopsy. This clinical presentation may be aspecific: abdominal pain and diarrhoea, intussusception or pain-simulating appendicitis; bleeding may be the only clinical manifestation of jejunum involvement, like patient number 1 of our series. Colon involvement from breast cancer can simulate a primary cancer of the colon or a Crohn's disease, both clinically and at imaging studies. Endoscopy with biopsy is mandatory to perform a definite diagnosis. Rectal involvement has also been described, including rectal stenosis. Oropharyngeal and esophageal involvement is rare but possible, presenting in general as dysphagia or pseudoachalasia [2, 8–25, 31]. 

A consistent finding in most reports is the overrepresentation of the infrequent lobular type of breast carcinoma in GI metastases [1, 61]. Several clinical and autopsy series [5, 83, 84] have examined the differences in the pattern of metastases of lobular and ductal breast carcinoma. A significantly higher prevalence of gastrointestinal and peritoneal metastases with lobular breast cancer has been found frequently; even in cases in which primary tumor is of mixed histology, it is the lobular component that tends to metastasize to the stomach [28, 33]. It has been suggested that ductal carcinomas tend to produce nodular stomach lesions, while lobular carcinomas tend to cause more diffuse disease [28]. According to the data of the literature, in two of our 5 cases the histology was LIC, two cases of DIC histology, and in 1 case it was carcinoma with signet ring cells. The patients' age varied, at the time of GI metastases diagnosis, from 40 to 75 years; the time from diagnosis of the primitive tumor to GI metastases raised from 0 to 22 years in our series, like in the literature, and we found that a case of gastric metastasis from breast cancer occurred 30 years after surgery for primary tumor [31].

In 4 out of 5 cases the diagnosis of GI relapse from breast cancer was made within few months (3-4) from symptoms' appearance; only in one case  18 months were needed to find gastric metastasis, and in this case a first EGDS with biopsies performed after symptoms' appearance was negative; this can be related to the submucosal infiltration of the metastatic tumor. It must be emphasised that endoscopy remains today the best diagnostic method to evaluate pathology of the upper gastrointestinal tract. However, we recall to the mind that in some conditions endoscopy can allow false negative results, that is, when the tumor is submucosal, and in these conditions endoscopy can be repeated or combined with endoscopic ultrasound (EUS). Reorientation of the ultrasound transducer on the echoendoscope allows for fine-needle aspiration capabilities, leading to increase of diagnostic accuracy in cancer diagnosis and staging [89].

In one case (jejunum localization) surgical intervention was necessary for the diagnosis and treatment.

All the 5 patients, at the time of GI relapse, had symptoms due to the site of relapse: inappetence, epigastralgia, dyspepsia, nausea, and vomiting (the patients affected by gastric relapse), and bleeding (the patient affected by localization to the jejunum and rectum).

 After reviewing the literature, data on treatment are fragmentary, and however patients underwent surgery when possible; the majority of the patients was treated with chemotherapy, one of them with endocrine therapy (especially after chemotherapy as maintenance therapy), and very rarely radiotherapy was performed. In 4 of 5 cases of our series, patients underwent chemotherapy, and 2 of them underwent subsequent maintenance hormonal therapy, whole one received also radiotherapy for rectal bleeding. One patient was treated only with hormonal therapy because of medical conditions suboptimal contraindicating chemotherapy. None of our patients was treated with trastuzumab since HER2 was not expressed.

Survival among reported cases in this paper varied from early deaths in patients reporting massive gastrointestinal bleeding to survival extended up to 44 months from the diagnosis of GI metastases [80]. In our series, according to the literature, the median overall survival after a diagnosis of GI metastasis is 16 months (range, 5–41 months), which is a little lower than the median survival of all women with metastatic disease secondary to breast cancer (range, 24–36 months) [86]. 

In conclusion, GI metastases are possible, even uncommon, and oncologists must consider it for patients affected by breast cancer, who develop GI symptoms, even if diagnosis and treatment for the primary tumour have been performed many years before. Adequate diagnostic procedures, like CT scan, endoscopy with biopsy, or EUS endoscopy with biopsy, must be performed as soon as possible, without delaying, to obtain a definite cytohistologic diagnosis allowing an adequate treatment for the patients.

## Figures and Tables

**Table 1:** Clinical characteristics of patients with breast cancer and GI tract involvement.

Pts/age	Breast cancer onset	Histology	TNM/G (breast cancer onset)	ER/PgR (%)	Ki-67 (%)	c erbB2	Treatment of primary tumor	Date and site of GI involvement	Treatment of GI involvement	Current status
I/47	March 1985	DIC	PT2N0M0/n.a.	>95/0	10	Negative	Surgery	December 2007/jejunum	Surgery, CT, ET	Alive June 2012
II/40	May 2008	Signet ring cells	CTxN1M1/2	90/35	15	Negative	CT, ET	May 2008/rectum	CT, ET, RT	Alive June 2012
III/53	1987	DIC	n.a./n.a.	>80/>80	10	Negative	Surgery	March 2008/stomach	CT	Death August 2008
IV/57	January 2006	LIC	PT2N3aM1/n.a.	80/85	10	Negative	Surgery, CT, ET	November 2008/stomach	CT	Death May 2009
V/63	1998	LIC	pT2N2M0/n.a.	50/0	10	Negative	Surgery, CT, ET	October 2010/stomach	ET	Alive June 2012

CT: chemotherapy; ET: endocrine therapy; RT: radiotherapy; DIC: ductal infiltrating carcinoma; LIC: lobular infiltrating carcinoma, n.a.: not assessable.

**Table 2:** Results from reviewing of the literature: patients with breast cancer and GI involvement.

Site of GI involvement	Clinical presentation/clinical symptoms	N. (%)	Treatment	References
Oropharynx	Oropharyngeal dysphagia	1	n.r.	[2]
	Tongue lesion	1	n.r.	[7]

Total oropharynx		2 (1%)		

Esophagus	Stricture	20	Dil., surg., ET	[2, 8–23]
	Achalasia	3	Surg., ET	[2, 24, 25]
	Nonspecific dysmotility	1	n.r.	[26]

Total esophagus		24 (12%)		

Stomach	Linitis plastica	57	Surg., CT, ET, RT	[2, 27–38]
	Obstruction/stenosis	27	Surg., CT	[1, 2, 27, 28, 39]
	Polyp	2	CT, ET, RT	[40, 41]
	Ulcer erosion/bleeding	12	CT, ET, RT, Surg.	[27, 28, 41–44]
	Perforation	2	Surg., CT, ET	[27, 45]
	Pain	10	CT, ET	[27, 46]
	Dyspepsia	11	n.r.	[27, 46]
	n.r.	1	n.r.	[47]
	n.r.	3	Surg., CT, ET	[48]

Total stomach		125 (60%)		

Small intestine	Obstruction/stenosis	12	Pall. care, surg., ET, RT, CT	[2, 49–57]
	Multiple obstructions	1	n.r.	[2]
	Peritonitis	1	Surg.	[58]
	Bleeding	1	Surg.	[59]
	n.r.	1	n.r.	[60]

Total small intestine		16 (8%)		

Colon	Obstruction/stenosis	16	ET, CT, RT, Surg.	[54, 61–69]
	Asymptomatic abdominal mass	1	Surg.	[1]
	Multiple strictures/diffuse infiltration	2	n.r.	[2, 70]
	Polyp	2	Surg., ET	[53, 71]
	Linitis plastica	1	Surg.	[72]
	n.r.	1	n.r.	[47]

Total colon		23 (11%)		

Rectum	Obstruction/stenosis	11	CT, RT, ET, Surg.	[56, 61, 73–77]
	Linitis plastica	1	n.r.	[33]
	Abdominal pain	1	CT	[39]
	Rectal bleeding	1	n.r.	[46]

Total rectum		14 (7%)		

Anum	Polyp	1	RT, ET	[78]
	n.r.	1	n.r.	[79]

Total anum		2 (1%)		

Total		206		

CT: chemotherapy; ET: endocrine therapy; RT: radiotherapy; Surg.: surgery; Dil: dilatation, Pall. care: palliative care; n.r.: not reported.
